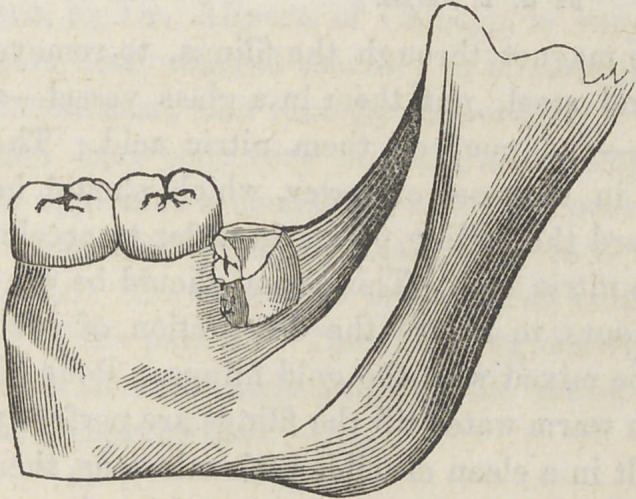# Richardson on Malposition

**Published:** 1857-09

**Authors:** J. Richardson


### MALPOSITION OF THIRD MOLAR.

BY J. RICHARDSON.

CASE.—Mr. J. T., aged thirty-five, called, complaining of pain in left inferior posterior molar. On examination, I found, just behind and resting upon the posterior face of this tooth, a small rounded portion of bone projecting about a line above the gum. This, on further exploration, proved to be a portion of the posterior face of a displaced or ante-verted wisdom tooth, lying parallel with the jaw, and resting closely with its grinding surface against the neck and roots of the second molar. The anterior half of the crown was deeply imbedded within the jaw, the processes running parallel with the long axis of the tooth. The root or roots passed directly back into the base of the coronoid process. There was nothing peculiar in the arrangement of the other teeth, if I may except some lateral contraction of the lower jaw and a somewhat crowded and irregular condition of the front teeth. The corresponding tooth of the opposite side had been extracted some years before.

We believe that this extreme displacement of these teeth is somewhat rare. An anterior or lateral *obliquity* is of frequent occurrence, but a crown standing directly at a right angle with the adjoining tooth is so seldom met with, that I have thought the case worthy of notice. In the Cabinet of the Mississippi Valley Association, I find a similar specimen contributed by Dr. Platon, of Troy, Ohio. These are the only two cases I have seen.

## Figures and Tables

**Figure f1:**